# The impact of cachexia index combined with BMI trajectory on survival outcomes in patients with cancer cachexia

**DOI:** 10.3389/fnut.2025.1706391

**Published:** 2026-01-08

**Authors:** Xue Cheng, Lijuan Guo, Junhao Ren, Jiaying Mo, Qing Li, Xin Jin, Yong Liu

**Affiliations:** 1 Xuzhou Clinical School of Xuzhou Medical University, Xuzhou, Jiangsu, China; 2 Department of Oncology, Xuzhou Central Hospital, Xuzhou Clinical School of Xuzhou Medical University, Xuzhou, Jiangsu, China

**Keywords:** cancer cachexia, cachexia index, BMI trajectory, subcutaneous fat thickness, survival prognosis, risk stratification

## Abstract

**Background:**

The cachexia index (CXI) has emerged as a recognized prognostic biomarker of cancer cachexia. However, the dynamic progression of cachexia may not be fully captured by a single assessment. This study examined the impact of integrating the CXI with BMI trajectories on the survival prognosis of patients with cancer cachexia to enable early identification of high-risk populations.

**Methods:**

This is a retrospective review of clinical and pathological data from 147 patients diagnosed with cancer cachexia at Xuzhou Central Hospital between January 2019 and May 2024. Based on computed tomography images at the time of initial cancer cachexia diagnosis to calculate the L3-SMI, the CXI was calculated using serum albumin (ALB) level, neutrophil-to-lymphocyte ratio (NLR), and skeletal muscle index (SMI). Using X-tile software, gender-specific optimal cutoff values for CXI were determined, and patients were divided into low and high CXI groups. Multiple BMI measurements were collected, and BMI dynamic trajectory subtypes were identified using latent category growth mixture modeling (GMM). Cox regression analysis was performed to identify independent risk factors for overall survival (OS); Kaplan–Meier survival curves were plotted; and subgroup interactions were analyzed according to cancer type, BMI trajectory subtype, ECOG PS, and TNM stage. A heterogeneity analysis of CT-based body composition was conducted to evaluate the relationship between muscle and adipose tissue.

**Results:**

GMM revealed two types of BMI decline trajectories: Class 1 (low reserve-slow decline BMI, 58%) and Class 2 (high reserve-accelerated decline BMI, 42%). The low CXI group had a considerably shorter median OS compared to the high CXI group (4.5 vs. 8.3 months, *p* < 0.001), with the low CXI and Class 1 subgroup having the poorest prognosis (median OS 3.5 months vs. other subgroups, *p* < 0.001). Multivariate Cox regression analysis identified low CXI, ECOG PS 2–3, and TNM Stage III-IV as independent risk factors for OS (*p* < 0.05). Subgroup analysis showed that low CXI significantly increased the risk of death in patients with gastrointestinal cancer, Class 1, and TNM Stage III-IV (*p* < 0.05), and there was no interaction with cancer type, BMI trajectory subtype, ECOG PS, or TNM Stage (*p* > 0.05). The third lumbar spinal muscle area (L3-SMA) was weakly positively correlated with subcutaneous fat thickness (SFT) (Spearman *r* = 0.256, *p* = 0.0018).

**Conclusion:**

The combination of low CXI and Class 1 trajectory was identified as an exceedingly high-risk phenotype with markedly poor survival, mandating early intensive intervention. This novel composite model provides a critical foundation for early risk stratification and precise intervention strategies in cancer cachexia, with the potential to significantly improve patient prognosis.

## Introduction

Cancer cachexia is a complex metabolic syndrome characterized by progressive skeletal muscle loss, with or without fat tissue reduction, directly impacting patient survival rates, treatment tolerance, and quality of life (1). It is estimated that approximately 50–80% of cancer patients may develop cachexia, with cachexia-related deaths accounting for over 20% of cancer-related mortality (2). Its pathogenesis fundamentally involves a tripartite dysregulation of muscle, inflammation, and metabolism (3, 4). While the international expert panel’s definition of cancer cachexia first explicitly identified persistent skeletal muscle loss as a core feature, its clinical application remains limited. Therefore, establishing a multidimensional biomarker model and implementing early intervention during cancer treatment are particularly crucial.

The Cachexia Index (CXI), initially developed to assess prognosis in non-small cell lung cancer patients (5), is calculated using three indicators: Skeletal Muscle Index (SMI), serum Albumin (ALB), and Neutrophil-to-Lymphocyte Ratio (NLR). Recent studies have confirmed its prognostic value across multiple malignancies (6–9). While BMI remains a core diagnostic indicator for cachexia as a baseline nutritional measure, static measurements fail to capture the complex and heterogeneous dynamic wasting patterns during cachexia progression (10). Additionally, high BMI may mask early-stage cachexia in obese patients (11). Growth mixture modeling (GMM) is a statistical method for analyzing individual trajectories over time. It groups individuals based on trajectory characteristics, where subgroups exhibit highly similar trajectories (intra-group homogeneity) and distinct trajectories between subgroups (inter-group heterogeneity) (12, 13). Therefore, this approach effectively addresses the limitations of single-time-point measurements.

This retrospective analysis of clinical and pathological data from cancer cachexia patients aims to investigate the combined impact of CXI and BMI trajectory subtypes on survival outcomes. The goal is to develop a more precise risk stratification model, providing a basis for early identification of high-risk patients and the formulation of personalized intervention strategies.

## Methods

### Study population

We retrospectively collected data on 147 patients diagnosed with cancer cachexia at Xuzhou Central Hospital between January 2019 and May 2024. The cohort comprised the following solid tumor types: lung cancer (*n* = 30), gastrointestinal cancer (*n* = 91), and other cancer (*n* = 26), the latter comprising head and neck, breast, and genitourinary cancers. The diagnostic criteria for cancer cachexia referenced the 2011 international expert consensus (1): (a) weight loss exceeding 5% within 6 months (excluding factors solely attributable to reduced food intake); (b) body mass index (BMI) < 20 kg/m^2^ and weight loss > 2%; (c) weight loss > 2% when diagnosed with sarcopenia. Meeting any one of the above three criteria was sufficient. The inclusion criteria were as follows: (a) age ≥ 18 years; (b) histologically or cytologically confirmed solid malignant tumor; (c) meeting Fearon criteria for cachexia diagnosis; (d) availability of laboratory and imaging data required for CXI calculation at initial cachexia diagnosis; (e) availability of at least three valid BMI measurements from initial cachexia diagnosis onwards. The exclusion criteria were as follows: (a) presence of severe, uncontrolled comorbid conditions; (b) concurrent acute infection; (c) diagnosis of hematologic malignancies; (d) incomplete clinical, pathological, or follow-up data. The data were collected from an electronic database. The study was conducted in accordance with the Declaration of Helsinki (revised in 2013) and was reviewed by the Medical Ethics Committee of our hospital (approval number: XZXY-LK-20241014-0154).

### Data collection

Clinical data were reviewed from electronic medical records at the time of initial diagnosis of cancer-related cachexia. The disease stage for all solid malignancies was classified according to the Tumor, Node, Metastasis (TNM) staging system of the American Joint Committee on Cancer (AJCC) Staging Manual, 8th edition. For the purpose of prognostic analysis, disease stage was categorized as early-stage (I–II) versus locally advanced or metastatic disease (III–IV). General information included: age, gender, height, weight, body mass index (BMI), Eastern Cooperative Oncology Group performance status (ECOG PS), Nutritional Risk Screening 2002 (NRS2002) score, comorbidities, and surgical history. Hematological indicators included: absolute neutrophil count, absolute lymphocyte count, and serum albumin. Imaging data included: Abdominal CT measurement of the third lumbar skeletal muscle area (SMA). Subcutaneous fat thickness (SFT) was measured at three abdominal sites: the linea alba and the mid-axillary lines on both sides, using the third lumbar vertebra level as the reference plane. The arithmetic mean of the three SFT measurements was used as the final abdominal subcutaneous fat thickness value (mm).

### Definitions

**Figure 1:**
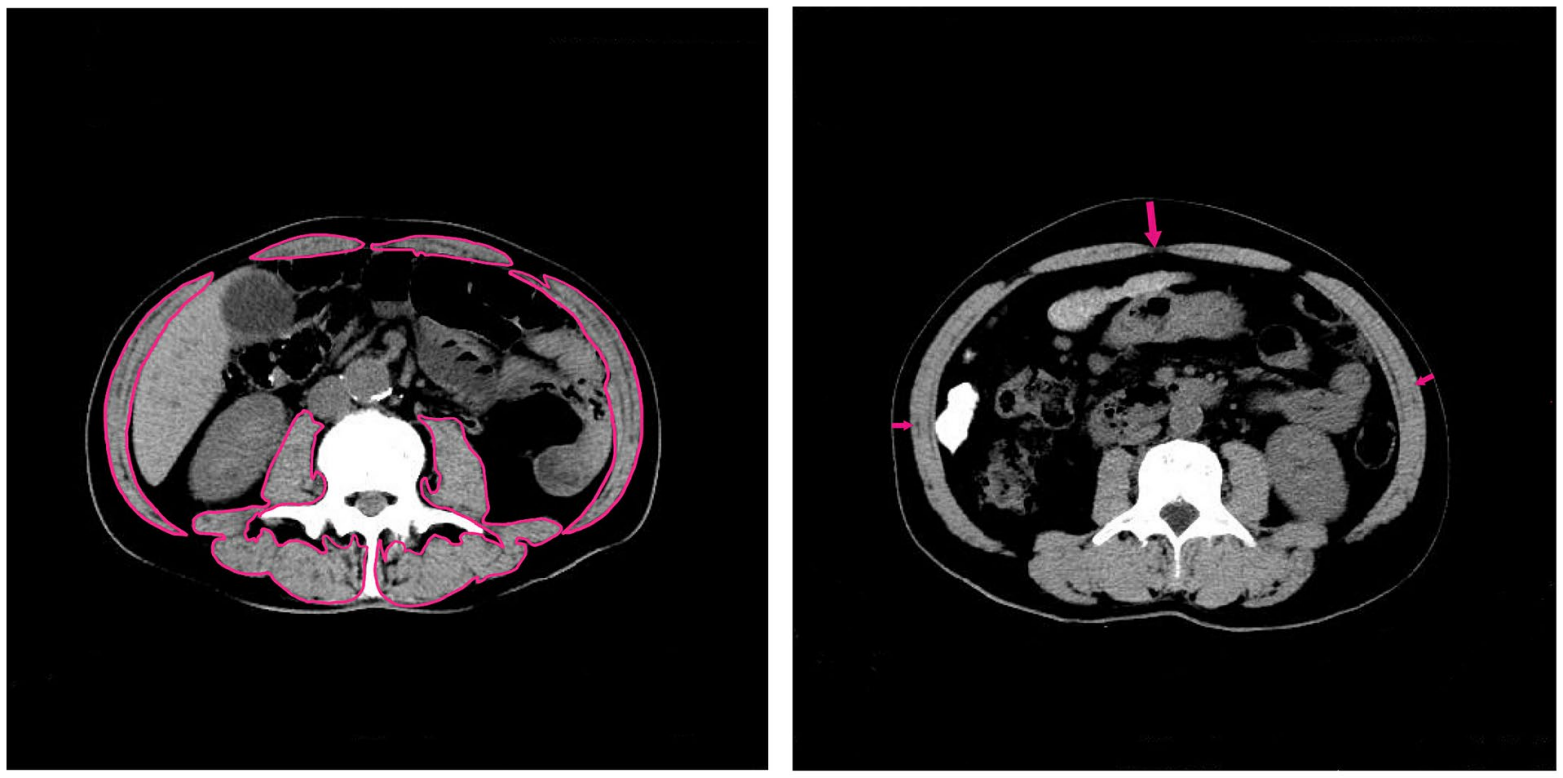
Schematic diagram of body composition evaluation. Measurements were performed on a single axial computed tomography (CT) image at the third lumbar vertebra (L3) level. The borders of all skeletal muscles were demarcated to quantify the skeletal muscle area (SMA). Subcutaneous fat thickness (SFT) was measured at three abdominal sites: the linea alba and bilateral mid-axillary lines. Tissue quantification was based on pre-defined Hounsfield unit thresholds (SMA: −29 to 150).

The primary endpoint of the study was overall survival (OS), defined as the time from initial diagnosis of cancer cachexia to the last follow-up or death from any cause. SMI was defined as the ratio of the total skeletal muscle area (cm^2^) at the L3 vertebral level on abdominal CT images at initial diagnosis of cancer cachexia to the square of the patient’s height (m^2^). At the L3 vertebral level, skeletal muscle was semi-automatically segmented via the PACS system, with Hounsfield unit (HU) thresholds defined as −29 to 150. After delineating all skeletal muscle boundaries within the L3 level, the system automatically quantified the total skeletal muscle area (Figure 1). NLR was defined as the ratio of absolute neutrophil count to absolute lymphocyte count. CXI was calculated using SMI (cm^2^/m^2^), serum albumin (g/dL), and NLR via the formula: CXI = SMI × ALB / NLR. BMI was defined as the ratio of a patient’s weight (kg) measured at different time points to the square of their height (m^2^). Weight data were sourced from inpatient medical records or outpatient follow-up records. Follow-up BMI data were collected after a diagnosis of cancer cachexia, with a minimum of three or more time points included.

### Statistical analysis

A growth mixture model (GMM) was constructed in Mplus 8.3 (14). Patients with at least three recorded BMI measurements were included to ensure sufficient longitudinal data for trajectory estimation. The model handled missing data under the missing-at-random (MAR) assumption using the Full Information Maximum Likelihood (FIML) method. A series of models, ranging from one to K classes, were sequentially fitted and compared using fit indices. The optimal BMI trajectory model was identified as that fulfilling the following criteria: minimized Bayesian Information Criterion (BIC), maximized entropy, an average posterior probability exceeding 70% for each trajectory group, and a minimum group size proportion greater than 5% (15). Data analysis was conducted using SPSS version 26.0. Normally distributed quantitative data are presented as mean ± standard deviation (SD) and compared between groups using independent samples *t*-tests. Non-normally distributed quantitative data are expressed as median (interquartile range) [M (QL, QU)] and compared across multiple groups using the Kruskal–Wallis test. Categorical data are summarized as number (%), and group comparisons were performed using chi-square (χ^2^) tests. The optimal cutoff value for the CXI for OS was calculated by selecting the minimum *p* value with the maximum chi-square value in all possible subdivisions of the populations using X-tile software. Kaplan–Meier survival curves were generated with GraphPad Prism 10.0, and survival times were compared via log-rank tests. The nonlinear association between L3-SMA and SFT was evaluated using Spearman rank correlation and visualized with locally weighted scatterplot smoothing (LOWESS) curves. Univariate and multivariate Cox regression models were used to analyze overall survival in patients with cancer cachexia. Subgroup analyses were performed based on cancer type, trajectory group, ECOG PS, and TNM Stage to assess the robustness of the Cox regression outcomes and to identify potential confounding factors. A two-sided *p* < 0.05 was considered statistically significant.

## Results

### Associations between L3-CXI and patient characteristics

Currently, there is no universally accepted cutoff value for the CXI internationally or in domestic practice. Using X-tile software, we calculated sex-specific optimal cutoff values based on survival data, which were 25.51 for males and 22.60 for females. Male patients with CXI > 25.51 and female patients with CXI > 22.60 were classified into the high CXI group (*n* = 91), while male patients with CXI ≤ 25.51 and female patients with CXI ≤ 22.60 were assigned to the low CXI group (*n* = 56). Additionally, the optimal survival-related cutoff values for NLR and SFT were 4.79 and 5.16, respectively. Compared to the high CXI group, patients in the low CXI group exhibited lower BMI, SMI, ALB, and SFT levels, higher NLR levels, and higher proportions of ECOG PS 2–3 and NRS 2002 ≥ 3 scores, with statistically significant differences (*p* < 0.05). No statistically significant differences were observed between the two groups in age, gender, cancer type, BMI trajectory group, TNM Stage, comorbidity, or surgery (*p* > 0.05, Table 1).

**Table 1:** Baseline characteristics of the two CXI groups.

Characteristics	Low CXI (*n* = 56)	High CXI (*n* = 91)	*p*
Age, *n* (%)			0.493
<60 years	19 (33.9)	45 (39.6)	
≥60 years	37 (66.1)	46 (60.4)	
Gender, *n* (%)			0.666
Male	33 (58.9)	49 (53.8)	
Female	23 (41.1)	42 (46.2)	
Type of cancer, *n* (%)			0.232
Lung cancer	13 (23.2)	17 (18.7)	
Gastrointestinal cancer	30 (53.6)	61 (67.0)	
Other cancer	13 (23.2)	13 (14.3)	
Trajectory group, *n* (%)			0.072
Class 1	37 (66.1)	45 (49.5)	
Class 2	19 (33.9)	46 (50.5)	
Median CXI (IQR)	13.8 (7.9, 19.0)	46.1 (34.4, 85.0)	**<0.001**
BMI, mean (SD), kg/m^2^	19.85 ± 2.79	21.51 ± 3.00	**0.001**
SMI, mean (SD), cm^2^/m^2^	32.24 ± 6.48	35.56 ± 6.57	**0.003**
Median NLR (IQR)	11.4 (6.3, 13.8)	2.8 (1.7, 3.7)	**<0.001**
ALB, mean (SD), g/dL	3.45 ± 0.53	3.88 ± 0.53	**<0.001**
SFT, mean (SD), mm	7.8 (5.1, 12.6)	9.9 (7.4, 14.0)	**0.013**
ECOG PS, *n* (%)			**<0.001**
0–1	14 (25.0)	53 (58.2)	
2–3	42 (75.0)	38 (41.8)	
NRS 2002, *n* (%)			**<0.001**
<3	10 (17.9)	51 (56.0)	
≥3	46 (82.1)	40 (44.0)	
TNM Stage, *n* (%)			1
I-II	8 (14.3)	13 (14.3)	
III-IV	48 (85.7)	78 (85.7)	
Comorbidity, *n* (%)			0.731
No	31 (55.4)	54 (59.3)	
Yes	25 (44.6)	37 (40.7)	
Surgery, *n* (%)			0.078
No	26 (46.4)	28 (30.8)	
Yes	30 (53.6)	63 (69.2)	

Class 1: “low reserve-slow decline BMI”; Class 2: “high reserve-accelerated decline BMI.”

CXI, cachexia index; BMI, body mass index; SMI, skeletal muscle mass index; ALB, serum albumin; NLR, neutrophil-lymphocyte ratio; SFT, subcutaneous fat thickness; ECOG PS, Eastern Cooperative Oncology Group performance status; NRS2002, Nutritional risk screening. The category “Other cancer” was composed of the following solid tumor types: head and neck, breast, and genitourinary cancers.

SD, Standard Deviation; Median (IQR), interquartile range, based on K-samples nonparametric test of association; *N* (%), based on *χ*^2^ test of association; Mean (SD), based on analysis of variance test of association. TNM staging was based on the 8th AJCC system. Bold values indicate statistically significant differences or associations (*p* < 0.05).

### Identification and determination of BMI trajectory

**Figure 2:**
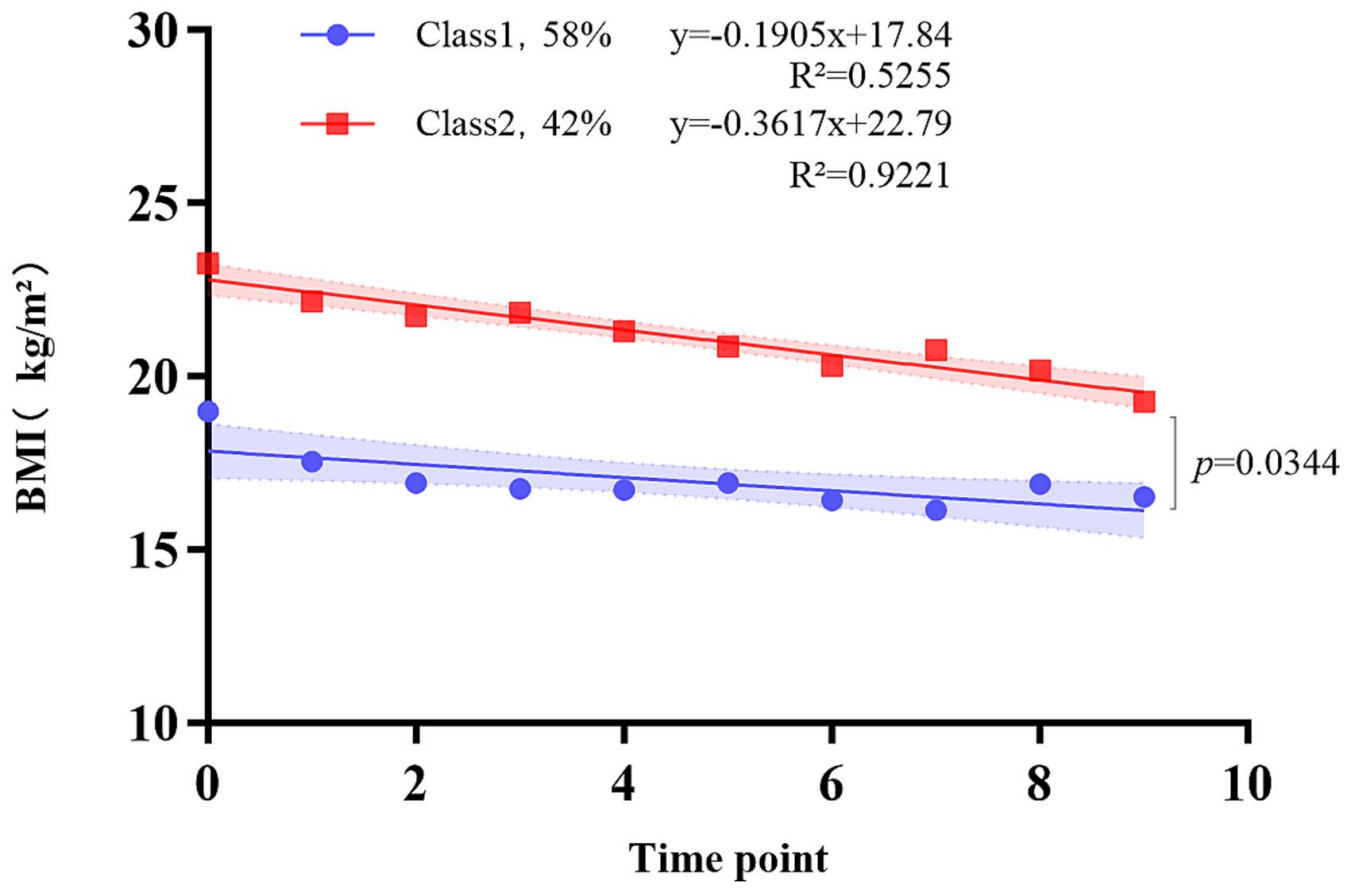
Model-estimated mean body mass index (BMI) trajectories for the two distinct classes identified by growth mixture modeling (GMM). Two patterns were revealed: class 1 (blue, *n* = 82), “low reserve–slow decline BMI” (slope: −0.191 kg/m^2^/time point), and class 2 (red, *n* = 65), “high reserve–accelerated decline BMI” (slope: −0.362 kg/m^2^/time point). The slope difference was significant (*p* = 0.034). Shadow shapes indicate 95% confidence interval.

Using BMI values at different time points as observational indicators, we included 147 patients with cancer cachexia in the model for analysis. GMM was employed to fit BMI trajectories from initial cachexia diagnosis to loss to follow-up or death across up to 10 predefined follow-up windows. The plotted trajectories represent the model-estimated means for each class. For missing values, the model typically applied the Full Information Maximum Likelihood (FIML) method, sequentially extracting three categories. When the number of latent categories was set to two, the BIC reached its minimum (3015.632), with a significant BLRT test (*p* = 0.0187), entropy >0.7, and a reasonable minimum class proportion, confirming it as the optimal BMI trajectory model, which can be found in Supplementary Table S1. After identifying two trajectory classes via the GMM model, linear regression was applied to standardized BMI changes at specific time points. Class 1 was defined as the “low reserve-slow decline” pattern, comprising 82 patients (58%) with a baseline mean BMI of 17.84 (95% CI: 17.06–18.63) and a decline rate of −0.191 per unit time point (SE: 0.064, *p* = 0.018). Class 2 was defined as the “high reserve-accelerated decline” pattern, comprising 65 patients (42%) with a baseline mean BMI of 22.79 (95% CI: 22.33–23.25) and a decline rate of −0.362 per time point (SE: 0.037, *p* < 0.001). The depletion rate in Class 2 was significantly faster than in Class 1 (slope difference: 0.171 kg/m^2^/point, 95% CI: 0.016–0.326, *p* = 0.034) (Figure 2).

### Survival analysis based on cachexia index and BMI trajectory subgroups

**Figure 3:**
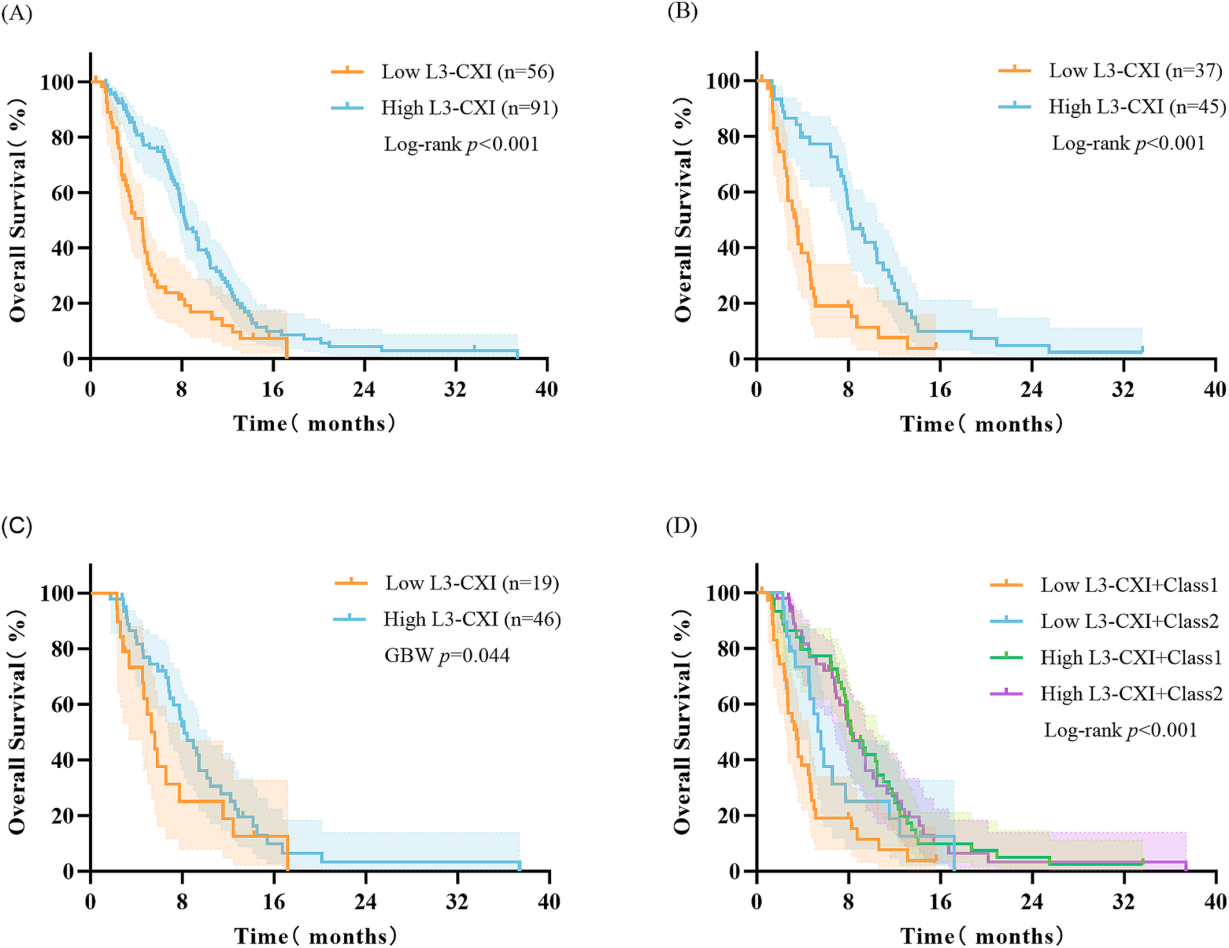
Kaplan–Meier survival analysis according to the level of L3-CXI. **(A)** OS in the entire cohort (*N* = 147) between high (*n* = 91) and low (*n* = 56) L3-CXI (log-rank *p* < 0.001). **(B)** OS stratified by L3-CXI within the Class 1 subgroup (*n* = 82; log-rank *p* < 0.001). **(C)** OS stratified by L3-CXI within the Class 2 subgroup (*n* = 65; GBW *p* = 0.044). **(D)** OS across the four combined subgroups defined by L3-CXI and BMI trajectory. Patients with low L3-CXI and Class 1 had the poorest prognosis (overall log-rank *p* < 0.001). CXI, cachexia index; OS, overall survival.

**Figure 4:**
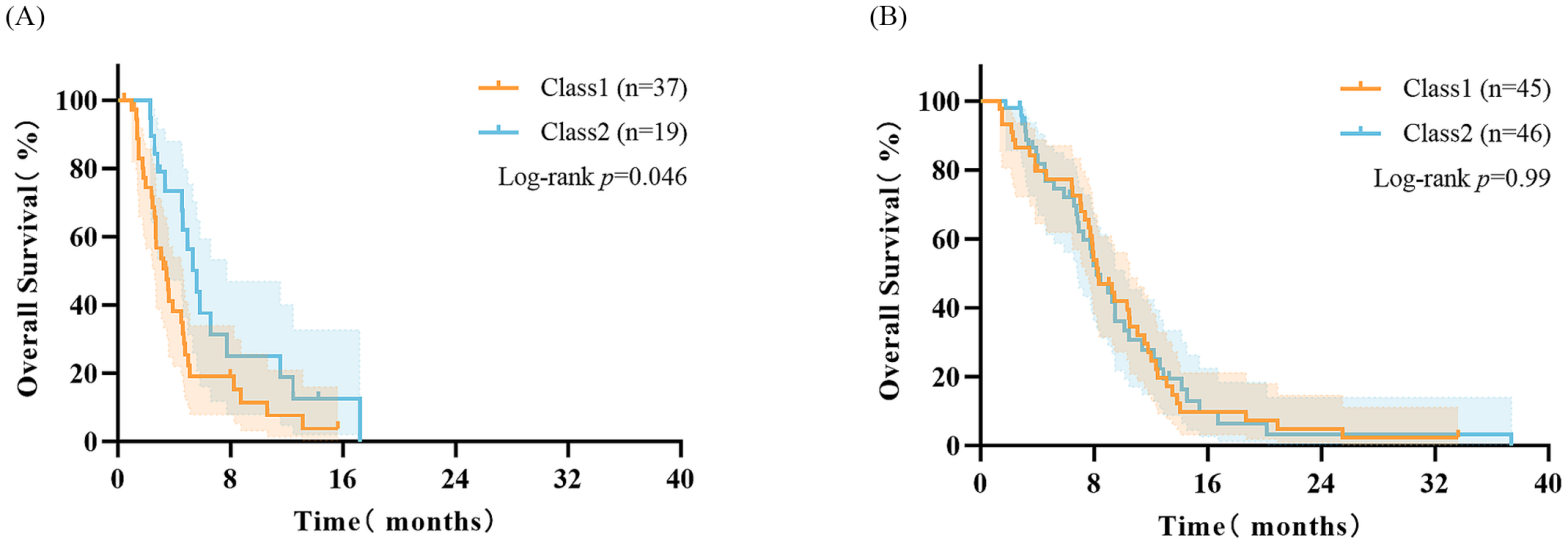
Impact of BMI trajectory on survival within L3-CXI subgroups. **(A)** OS by BMI trajectory (Class 1 vs. Class 2) in the low L3-CXI subgroup (*n* = 56; Log-rank *p* = 0.046). **(B)** OS by BMI trajectory (Class 1 vs. Class 2) in the high L3-CXI subgroup (*n* = 91; Log-rank *p* = 0.990). CXI, cachexia index; OS, overall survival.

The high CXI group demonstrated survival advantages in both the overall population and BMI trajectory subgroups: Median OS in the overall population (8.3 months vs. 4.5 months, log-rank *p* < 0.001, Figure 3A), median OS in Class 1 (8.3 months vs. 3.5 months, log-rank *p* < 0.001, Figure 3B), median OS in Class 2 (8.2 months vs. 5.6 months, GBW *p* = 0.044, Figure 3C). When CXI was combined with BMI trajectory, the low CXI and Class 1 subgroup had the poorest prognosis with a median OS of only 3.5 months (vs other subgroups, *p* < 0.001, Figure 3D). The low CXI subgroup showed a poor overall survival (median OS < 8 months), but a statistically significant difference was observed between Class 1 and Class 2 within this subgroup (*p* < 0.05, Figure 4A). The high CXI subgroup showed a significant survival advantage (median OS > 8 months), but no statistically significant difference was observed between Class 1 and Class 2 within this subgroup (*p* > 0.05, Figure 4B).

### Prognostic value of CXI in early-stage Cancer

To explore the applicability of our model across the disease spectrum, a sensitivity analysis was conducted on the 21 patients with early-stage (I-II) cancer. In this subgroup, patients with low CXI showed a strong trend toward poorer survival (5.1 months vs. 12.0 months, log-rank *p* = 0.139, HR: 2.355, Supplementary Figure S1).

### Univariate and multivariate analysis of clinicopathological variables in relation to OS in patients with cancer cachexia

**Figure 5:**
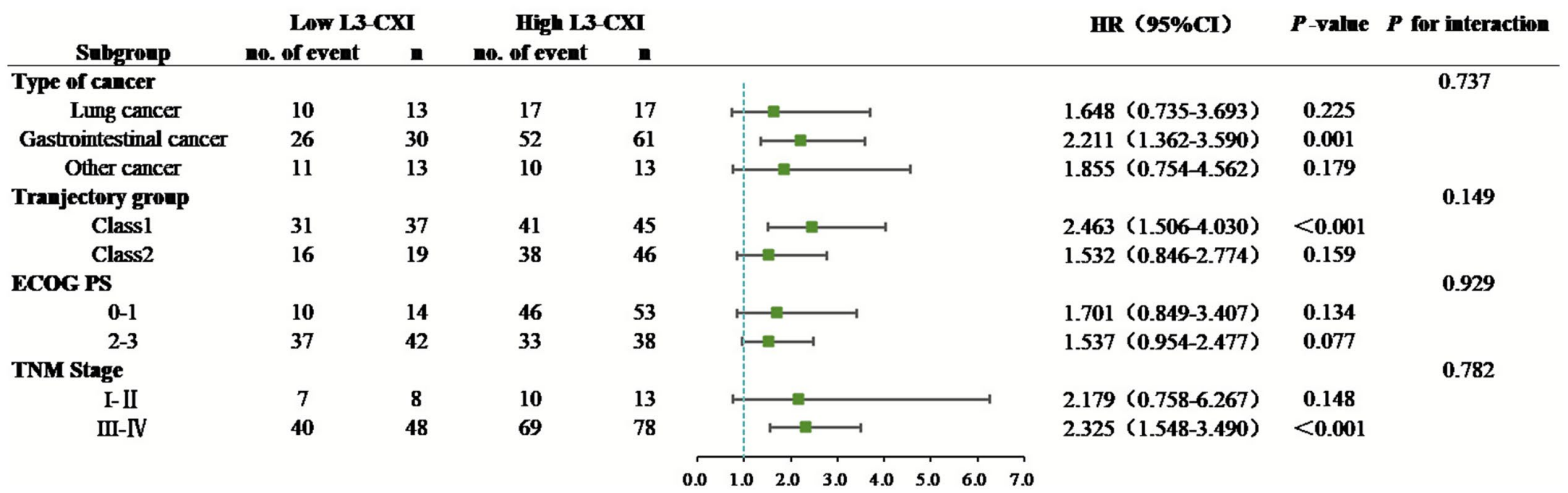
Forest plot of subgroup analysis for the association between L3-CXI and overall survival in patients with cancer cachexia. Hazard ratios (HR) and 95% confidence intervals (CI) are derived from univariable Cox regression within each subgroup. No significant interactions were observed (all *p*-interaction > 0.05), indicating the consistent prognostic value of L3-CXI. CXI, cachexia index; Class1: “low reserve-slow decline BMI”; Class2: “high reserve-accelerated decline BMI”; ECOG PS, Eastern Cooperative Oncology Group performance status; TNM staging was based on the 8th AJCC system.

Univariate analysis revealed that age, CXI, SMI, serum ALB, NLR, ECOG PS, TNM Stage, and surgery were significantly associated with overall survival (OS) in patients with cancer cachexia (*p* < 0.05, Table 2). Variables with *p* < 0.05 were included in the multivariate Cox regression. To avoid multicollinearity, the composite biomarker CXI was selected for inclusion over its individual components (SMI, ALB, and NLR); a direct model comparison confirming the superior stability and prognostic integrity of CXI is provided in Supplementary Table S2. The final multivariate model, which exhibited no significant collinearity (all variance inflation factors [VIFs] < 2), identified low CXI (HR: 1.749, 95% CI: 1.165–2.625, *p* = 0.007), ECOG PS 2–3 (HR: 2.262, 95% CI: 1.505–3.400, *p* < 0.001), and TNM Stage III-IV (HR: 2.281, 95% CI: 1.303–3.991, *p* = 0.004) as independent risk factors for OS (Table 2). Subgroup analysis revealed no significant interactions between cancer type, BMI trajectory group, ECOG PS, TNM Stage, and CXI (*p* > 0.05). However, the risk of death was increased in all prespecified subgroups with low CXI (HR > 1). The most significant risk increase was observed in patients with low CXI in gastrointestinal cancer (HR = 2.211, *p* = 0.001), Class 1 trajectory group (HR = 2.463, *p* < 0.001), and patients with TNM Stage III-IV (HR = 2.325, *p* < 0.001) (Figure 5).

**Table 2:** Univariate and Multivariate Cox regression analysis for overall survival in patients with cancer cachexia.

Characteristics	Univariate analysis		Multivariate analysis	
	HR (95% CI)	*p*	HR (95% CI)	*p*
Age (≥60 vs. < 60, years)	1.510 (1.042–2.186)	**0.029**	1.175 (0.811–1.702)	0.394
Gender (Male vs. Female)	1.157 (0.812–1.649)	0.420		
Type of cancer				
LC vs. Others	0.844 (0.473–1.505)	0.564		
GIC vs. Others	0.664 (0.407–1.084)	0.101		
Trajectory group (Class1 vs. Class2)	1.215 (0.851–1.734)	0.283		
CXI (Low vs. High)	2.072 (1.428–3.007)	**<0.001**	1.749 (1.165–2.625)	**0.007**
BMI (<18.5 vs. ≥ 18.5, kg/m^2^)	1.147 (0.739–1.779)	0.541		
SMI (Low vs. High)	1.975 (1.365–2.858)	**<0.001**		
ALB (<35 vs. ≥ 35, g/L)	2.130 (1.464–3.101)	**<0.001**		
NLR (>4.79 vs. ≤ 4.79)	1.657 (1.150–2.386)	**0.007**		
SFT (≤5.16 vs.>5.16)	1.493 (0.924–2.414)	0.102		
ECOG PS (2–3 vs. 1–2)	2.608 (1.792–3.796)	**<0.001**	2.262 (1.505–3.400)	**<0.001**
TNM Stage (III-IV vs. I-II)	2.067 (1.208–3.536)	**0.008**	2.281 (1.303–3.991)	**0.004**
Comorbidity (No vs. Yes)	1.143 (0.797–1.640)	0.468		
Surgery (No vs. Yes)	1.478 (1.025–2.131)	**0.037**	1.219 (0.834–1.782)	0.307

LC, lung cancer; GIC, gastrointestinal cancer; HR, hazard ratio; CI, confidence interval; Class 1: “low reserve-slow decline BMI”; Class 2: “high reserve-accelerated decline BMI.”

CXI, cachexia index; BMI, body mass index; SMI, skeletal muscle mass index; ALB, serum albumin; NLR, neutrophil-lymphocyte ratio; SFT, subcutaneous fat thickness; ECOG PS, Eastern Cooperative Oncology Group performance status; TNM staging was based on the 8th AJCC system. Bold values indicate statistically significant differences or associations (*p* < 0.05).

### Heterogeneity analysis of CT body composition

**Figure 6:**
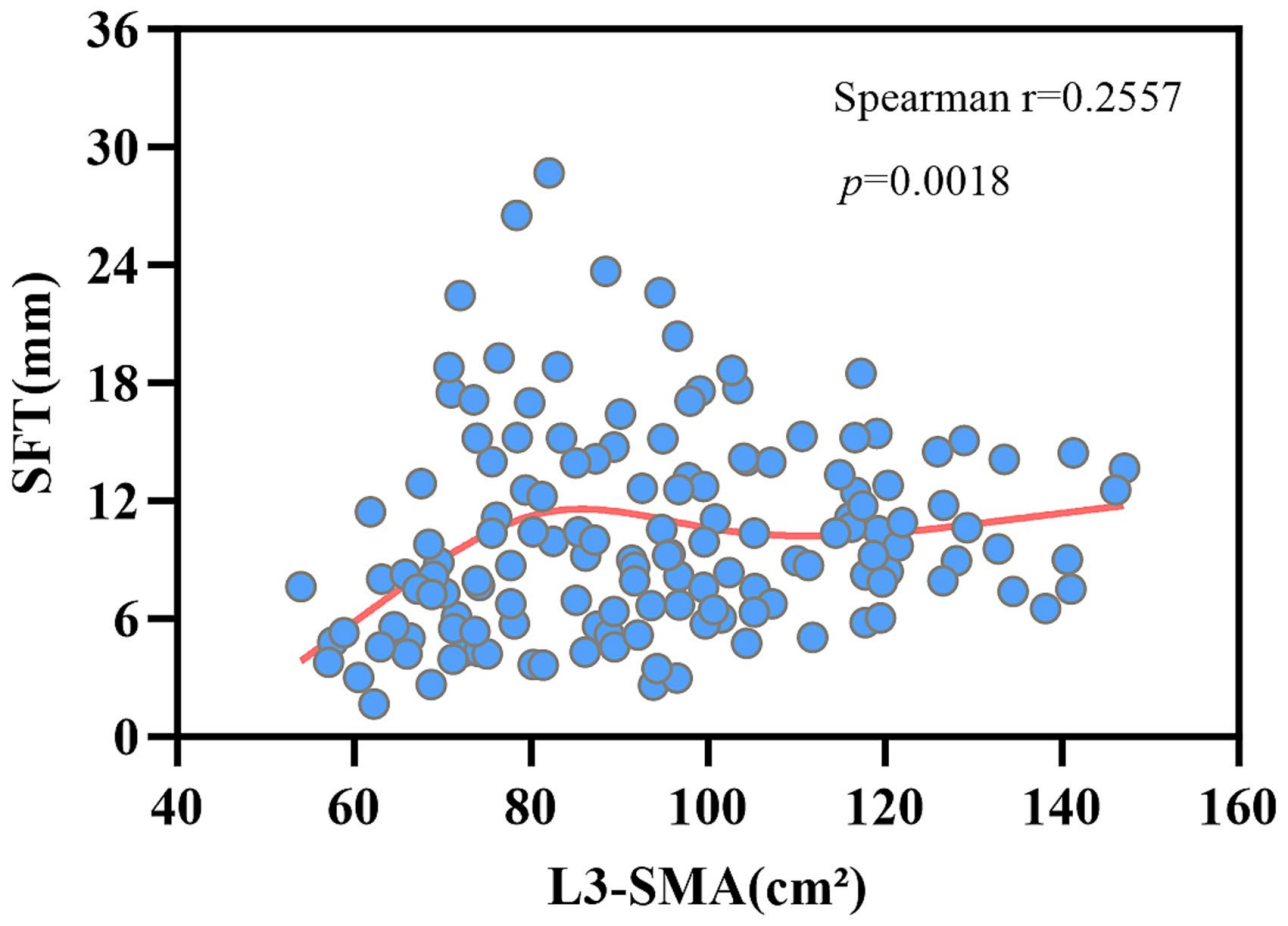
Scatter plot of subcutaneous fat thickness (SFT) versus L3 skeletal muscle area (SMA) in cancer cachexia patients at baseline. A significant weak positive correlation (Spearman *r* = 0.256, 95% CI: 0.093–0.405, *p* = 0.0018) supported the heterogeneity in wasting trajectories.

The lumbar skeletal muscle area at L3 (L3-SMA) showed a significant weak positive correlation with subcutaneous fat thickness (SFT) (Spearman r = 0.256, 95% CI: 0.093–0.405, *p* = 0.0018). The scatter plot revealed a nonlinear distribution pattern (Figure 6). CT body composition quantification confirmed the asynchronous consumption of muscle and fat during the process of cancer cachexia, providing imaging evidence for the heterogeneity of BMI dynamic trajectories.

### Association of BMI trajectories with baseline body composition

To investigate the morphological basis of the distinct BMI trajectories, we compared baseline body composition between Class 1 and Class 2 groups. Patients in the Class 2 trajectory had significantly greater baseline reserves of both skeletal muscle and adipose tissue compared to those in Class 1, as evidenced by higher L3-SMA (99.5 vs. 82.3 cm^2^, *p* < 0.001) and SFT (12.5 vs. 7.3 mm, *p* < 0.001) (Table 3).

**Table 3:** Comparison of baseline body composition between BMI trajectory groups.

Variable	Class 1 (*n* = 82)	Class 2 (*n* = 65)	*p*
L3-SMA (cm^2^)	82.3 (70.6,99.9)	99.5 (86.7,118.9)	<0.001
SFT (mm)	7.3 (5.0,10.7)	12.5 (9.0,15.2)	<0.001

Data are presented as median (interquartile range). Comparisons between groups were made using the Mann–Whitney U test. Class 1: “low reserve-slow decline BMI”; Class 2: “high reserve-accelerated decline BMI”; L3-SMA, the third lumbar skeletal muscle area; SFT, subcutaneous fat thickness.

## Discussion

In recent years, multiple studies have demonstrated that nutritional risk is closely associated with poor prognosis in cancer patients. Clinically, weight loss is primarily used as a quantitative screening criterion for cachectic patients. However, significant individual variation exists among cachectic patients, particularly in advanced cancer patients, where characteristics such as malignant ascites or large masses often mask actual weight loss. This failure to accurately reflect the patient’s actual nutritional status can lead to missed or misdiagnoses to some extent (16). Although the NRS2002 can identify cancer patients with nutritional risks, it relies on patient-reported appetite or intake, which is highly subjective. Moreover, it cannot quantify muscle loss and inflammatory burden, and has limited value in the early diagnosis of cachexia (17). Therefore, there is an urgent need to integrate objective indicators to enhance diagnostic efficacy. Although the continuous loss of skeletal muscle mass is a crucial manifestation and marker of cancer cachexia, there is no practical method for measuring whole-body skeletal muscle mass at present. Mourtzakis M et al. (18) found that the ratio of skeletal muscle and adipose tissue at the L3 vertebral level highly correlates with the whole-body ratio of skeletal muscle and adipose tissue. Consequently, the SMI at the L3 vertebral level has been used to evaluate skeletal muscle quantity (19). Jafri et al. (5) pioneered the CXI as a composite metric encompassing the three primary clinical features of cancer cachexia: malnutrition, systemic inflammation, and sarcopenia (20–22). Each of the features has been demonstrated in prior studies to correlate closely with patient prognosis. Among them, sarcopenia constitutes the most critical component of the CXI. Its primary driving mechanism involves cytokine-mediated acceleration of proteolytic catabolism (23). Sarcopenia not only reflects deteriorating nutritional status and disease progression but also significantly reduces tolerance to anticancer treatments, resulting in further disease worsening and poor prognosis (24, 25). Previous research predominantly used median values to divide the CXI into groups without considering the impact of gender differences on grouping results. Given the positive correlation between CXI and SMI, as well as the inherent gender specificity of the SMI cutoff value for diagnosing sarcopenia, this study used X-tile software to establish gender-specific CXI cutoff values, thereby making the grouping more reasonable.

Previous studies have primarily validated the prognostic value of CXI in single cancer types such as diffuse large B-cell lymphoma, gastric cancer, and ovarian cancer (6, 8, 26). This study confirms in a mixed-cancer cohort that low CXI is an independent risk factor for overall survival in cancer cachexia patients. Additionally, patients with low CXI exhibit clinical and pathological characteristics, including lower BMI and SMI, reduced serum ALB levels, higher NLR, and a higher proportion of TNM Stage III-IV disease. This finding aligns strongly with Xu et al.’s meta-analysis (27), indicating that CXI, as a core biomarker reflecting the “muscle-inflammation-metabolism” triad imbalance, may demonstrate broad applicability across diverse cancer types.

Our application of GMM to analyze BMI trajectories in cancer cachexia patients, which revealed two distinct subgroups with divergent clinical characteristics, is supported by growing evidence underscoring the importance of longitudinal assessment. This approach is powerfully validated by the recent work of Zhi-Cheng et al. (28), who used serial CT-based body composition analysis to identify distinct cachexia phenotypes with differential survival outcomes, affirming the critical value of dynamic wasting patterns over single-timepoint assessments. Our study demonstrates that dynamic BMI trajectories, a more universal and clinically actionable metric, can effectively identify patient subgroups with comparable prognostic significance. By integrating these dynamic trajectories with the static CXI, we have developed a composite model that synthesizes precise phenotyping with scalable clinical application.

Despite higher baseline BMI in Class 2 patients (22.79 vs. 17.84 kg/m^2^), their wasting rate was 1.9 times that of Class 1 patients, with median OS of only 5.6 months in the low CXI subgroup. This finding offers a novel interpretation of the “obesity paradox” (29): elevated BMI may mask rapid muscle loss, and traditional nutritional screening often misses Class 2 patients. Mechanistically, the high reserve-accelerated decline phenotype in Class 2 patients aligns closely with previously reported metabolic crisis characteristics (3) —lactic acid accumulation in the tumor microenvironment activates adipose tissue GPR81 receptors, triggering fat browning and muscle breakdown via the Gi-Gβγ-RhoA/ROCK1-p38 signaling axis. Notably, this mechanism primarily occurs in patients with normal baseline reserves who experience sudden rapid depletion. In contrast, the Class 1 phenotype, characterized by low reserve and slow decline, is more likely associated with sustained chronic inflammatory factor activation (30).” The worst prognosis in this study was observed in the low CXI and Class 1 group (median OS only 3.5 months), highlighting the combined effect of depleted physiological reserves and persistent depletion. The high CXI group (regardless of Class 1 or Class 2) consistently demonstrated survival advantages (median OS > 8 months), with no statistically significant differences in trajectories within the group (*p* > 0.05). This advantage likely stems from the physiological protective effect of high CXI, which can be explained in three aspects: First, the triple physiological reserves of high CXI (superior muscle reserve, favorable nutritional metabolism, and a low systemic inflammatory burden) buffer against short-term depletion risks; Second, accelerated depletion in Class 2 requires reaching a specific threshold to manifest survival differences. Xie et al. (31) suggest that the clinical threshold for weight loss increases with rising BMI, and reserves compensate before threshold attainment. However, some patients experience a short disease duration from cachexia diagnosis to death, with depletion processes not yet reaching this critical threshold. Third, patients with high CXI demonstrate superior response to systemic therapy (32)^–^ (33), where treatment benefits partially offset depletion risks. Thus, the combined model quantifies immediate physiological reserves via static CXI metrics and metabolic collapse risk via dynamic BMI trajectories. It not only identifies the population with terminal cachexia (low CXI and Class 1) but also alerts to metabolic risks in high-reserve individuals (Class 2). Although the latter group maintains short-term survival under high CXI buffering without damage, their accelerated depletion signals an opportunity for an intervention window. Metabolically targeted interventions should be explored during the compensatory phase rather than relying solely on nutritional support. The synergistic use of both indicators enhances the accuracy of risk stratification compared to single-marker approaches (10).

This study quantitatively analyzed L3-SMA and SFT using single-scan CT imaging, revealing only a weak correlation between the two (Spearman r = 0.256, *p* = 0.0018) with a nonlinear scatter plot distribution, indicating asynchronous depletion of these tissues. Critically, the comparative analysis between trajectory groups revealed that these phenotypes are rooted in distinct baseline body composition states. Specifically, the Class 2 phenotype was objectively defined by significantly greater baseline reserves of both L3-SMA and SFT compared to Class 1. This finding clarifies that the divergent BMI trajectories are not merely differences in depletion speed, but are pre-determined by the patient’s initial morphological status. The subsequent rapid decline in Class 2 likely represents a catabolic state that aggressively consumes these substantial reserves, potentially driven by the metabolic imbalance mechanisms mentioned elsewhere (3). In contrast, the Class 1 phenotype, starting from a state of comprehensively depleted reserves, is more likely associated with a slower, chronic consumptive process under sustained inflammatory activation (34).

Despite these novel findings, several limitations should be considered. First, the retrospective, single-center design carries inherent risks of selection bias, and the sample size may limit the generalizability of our findings. For instance, a sensitivity analysis in our early-stage subgroup (*n* = 21) revealed a concordant prognostic trend for CXI but was underpowered to formally validate the model or to analyze BMI trajectories. Furthermore, the diagnosis of cachexia was based on the 2011 international consensus criteria, which was the most feasible for our retrospective design. Future prospective studies would benefit from incorporating newer frameworks, such as the AWGC 2023 criteria (35), to include critical dimensions like grip strength and patient-reported outcomes. Therefore, multicenter, prospective studies encompassing patients across all disease stages, including those with early-stage cancer, are warranted to validate the utility of this composite model. Regarding survival curve crossovers within the high CXI group, if this phenomenon persists after expanding the sample size, landmark analysis can be employed to segment evaluations based on “reaching the critical threshold for depletion” (36). Future studies may incorporate metabolic biomarkers (e.g., lactate) to validate the metabolic imbalance mechanisms in Class 2 patients and explore potential interventions targeting these pathways (e.g., GPR81 inhibitors). Finally, as baseline CT imaging alone cannot capture dynamic changes, Future studies should integrate multiple longitudinal CT scans to quantify temporal rates of muscle and adipose tissue depletion across trajectory groups, thereby clarifying distinct catabolic patterns in Class 1 versus Class 2 phenotypes.

## Conclusion

In conclusion, CXI is a valuable biomarker for cancer cachexia that has a high prognostic significance. We have developed the first precision-stratification model for predicting outcomes in cancer cachexia patients by creatively combining static CXI with dynamic BMI trajectories. The “high reserve-accelerated decline” phenotype exhibited by the Class 2 trajectory represents an early signal of metabolic imbalance, with its core clinical significance lying in providing an early warning window. Based on these findings, we propose the following dynamic management pathways: For patients with high CXI and Class 2 trajectory, intensified monitoring of metabolic status (e.g., regular assessment of metabolic markers, such as lactate) is warranted, alongside proactive exploration of the optimal timing for metabolic interventions. For patients with low CXI and Class 1 trajectory, palliative care and symptom management should be prioritized to enhance quality of life. Prospective studies are needed to validate the feasibility of this approach. Such efforts could facilitate a paradigm shift in cachexia management—from reactive support to proactive, early-warning, and stratified intervention—potentially improving overall survival and quality of life in patients with cancer cachexia.

## Data Availability

The original contributions presented in the study are included in the article/Supplementary material, further inquiries can be directed to the corresponding author.
