# Supplementary material for: The impact of cachexia index combined with BMI trajectory on survival outcomes in patients with cancer cachexia
Source: Front Nutr. 2026 Jan 8;12:1706391. doi: 10.3389/fnut.2025.1706391 (PMC12823498; doi:10.3389/fnut.2025.1706391)
Supplement: Supplementary file 1 [file Table_1.DOCX]

**Supplementary Table S1** Model fitting parameters

Abbreviations: BIC, Bayesian Information Criterion; AIC, Akaike Information Criterion; aBIC, Sample-corrected BIC; LMR, Likelihood Ratio Test; BLRT, Bootstrap-based Likelihood Ratio Test.

**Supplementary Table S2** Comparison of the Composite Cachexia Index (CXI) and Its Individual Components in Multivariate Cox Regression Models

| Variable | Model A | |  | Model B | |
| --- | --- | --- | --- | --- | --- |
|  | HR (95%CI) | *P* |  | HR (95%CI) | *P* |
| Age（≥60 vs <60, years） | 1.175(0.811-1.702) | 0.394 |  | 1.144(0.779-1.680) | 0.494 |
| CXI（Low vs High） | 1.749(1.165-2.625) | **0.007** |  |  |  |
| SMI（Low vs High） |  |  |  | 0.995(0.683-1.452) | 0.981 |
| ALB（＜35 vs ≥35, g/L） |  |  |  | 1.774(1.185-2.656) | **0.005** |
| NLR（＞4.79 vs ≤4.79） |  |  |  | 1.148(0.765-1.725) | 0.505 |
| ECOG PS（2-3 vs 1-2） | 2.262(1.505-3.400) | **<0.001** |  | 2.243(1.485-3.389) | **<0.001** |
| TNM Stage（Ⅲ-Ⅳ vs Ⅰ-Ⅱ） | 2.281(1.303-3.991) | **0.004** |  | 2.094(1.193-3.674) | **0.010** |
| Surgery（No vs Yes） | 1.219(0.834-1.782) | 0.307 |  | 1.313(0.894-1.928) | 0.164 |

Notes: Model A includes the composite CXI. Model B includes the individual components of CXI (SMI, ALB, NLR) without CXI itself. Both models are adjusted for Age, ECOG PS, Stage, and Surgery. Abbreviations: CXI, cachexia index; SMI, skeletal muscle mass index; ALB, serum albumin; NLR, neutrophil-lymphocyte ratio; ECOG PS, Eastern Cooperative Oncology Group performance status; TNM staging was based on the 8th AJCC system; CI, confidence interval; HR, hazard ratio.

**Supplementary Figure S1**. Kaplan-Meier survival analysis for the early-stage subgroup (TNM Stage I-II, n=21) stratified by the CXI.


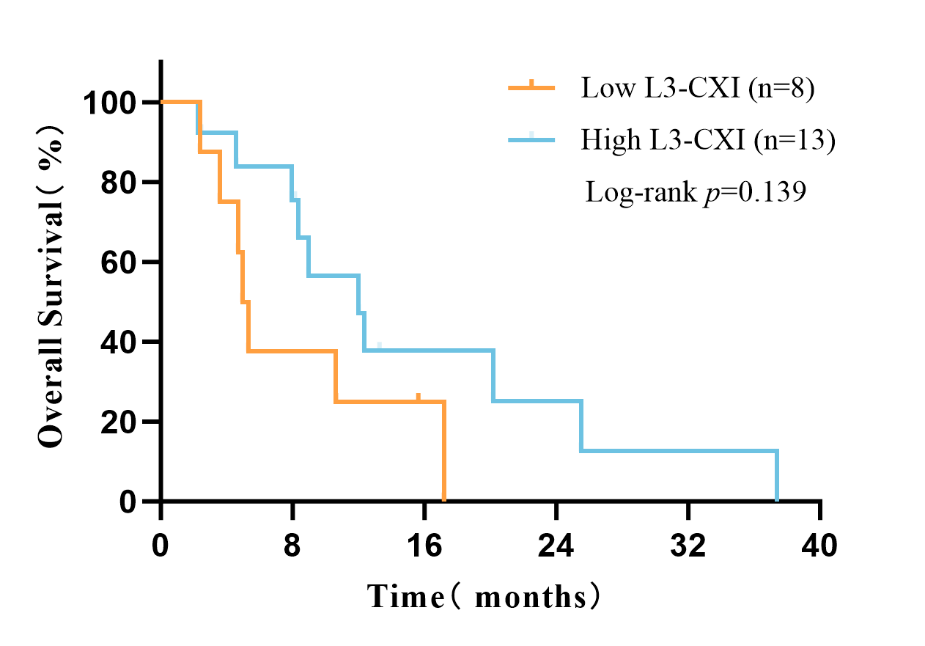


Abbreviations: CXI, cachexia index; OS, overall survival.
